# 3β-Corner Stability by Comparative Molecular Dynamics Simulations

**DOI:** 10.3390/ijms231911674

**Published:** 2022-10-02

**Authors:** Vladimir R. Rudnev, Kirill S. Nikolsky, Denis V. Petrovsky, Liudmila I. Kulikova, Anton M. Kargatov, Kristina A. Malsagova, Alexander A. Stepanov, Arthur T. Kopylov, Anna L. Kaysheva, Alexander V. Efimov

**Affiliations:** 1 Biobanking Group, Branch of Institute of Biomedical Chemistry “Scientific and Education Center”, 109028 Moscow, Russia; 2 Institute of Theoretical and Experimental Biophysics, Russian Academy of Sciences, 142290 Pushchino, Russia; 3 Institute of Protein Research, Russian Academy of Sciences, 142290 Pushchino, Russia

**Keywords:** super-secondary structure, 3β-corner, folding nuclei, structure stability

## Abstract

This study explored the mechanisms by which the stability of super-secondary structures of the 3β-corner type autonomously outside the protein globule are maintained in an aqueous environment. A molecular dynamic (MD) study determined the behavioral diversity of a large set of non-homologous 3β-corner structures of various origins. We focused on geometric parameters such as change in gyration radius, solvent-accessible area, major conformer lifetime and torsion angles, and the number of hydrogen bonds. Ultimately, a set of 3β-corners from 330 structures was characterized by a root mean square deviation (RMSD) of less than 5 Å, a change in the gyration radius of no more than 5%, and the preservation of amino acid residues positioned within the allowed regions on the Ramachandran map. The studied structures retained their topologies throughout the MD experiments. Thus, the 3β-corner structure was found to be rather stable per se in a water environment, i.e., without the rest of a protein molecule, and can act as the nucleus or “ready-made” building block in protein folding. The 3β-corner can also be considered as an independent object for study in field of structural biology.

## 1. Introduction

Structural motifs (super-secondary structures, SSS) of globular proteins are defined as commonly occurring folding units composed of two or more elements of secondary structure that are adjacent along the polypeptide chain and are in close contact in three-dimensional space. While many different structural motifs have been observed to recur within proteins, only some of motifs exhibit the definite handedness and a unique overall fold, irrespective of whether they occur in homologous or non-homologous proteins [1,2,3,4]. The high incidence of occurrence of structural motifs in unrelated proteins and the fact that many small proteins are merely composed of the motifs indicate their stability and ability to fold into unique structures per se. Such structural motifs are of particular interest since they can act as nuclei, or “ready-made” building blocks, in protein folding, or can be utilized as starting structures in protein modeling [4,5]. The structural motif with a unique overall fold occurring over all proteins of the structural group can be taken as the starting structure in modeling or as the root structure of structural tree. Larger protein structures are obtained by stepwise addition of α-helices and/or β-strands to the root motif in accordance with a restricted set of rules inferred from known principles of protein structures. Several structural trees for protein superfamilies, including several thousand of known proteins (their 3D structures were taken from PDB), have been constructed and are available at the following source: http://strees.protres.ru/ (accessed on 15 August 2022).

There is growing interest in structural motifs because they can be embryos in the process of protein folding. Over the past 30 years, significant research efforts have been made to identify and characterize the folding nuclei for the known protein structures [6,7]. In this scope, structural motifs were broadly discussed as a promising candidate [7,8,9]. The prominent representatives of structural motives are α-α-corner, β-hairpin, and Greek key motif [10]. Specification of these structures caused researchers to develop a catalog of autonomously folded protein motifs and archetypes.

This study is devoted to comparative analysis of variety molecular dynamics simulations of the stability of 3β-corner structural motif (the super-secondary structure).

**Figure 1:**
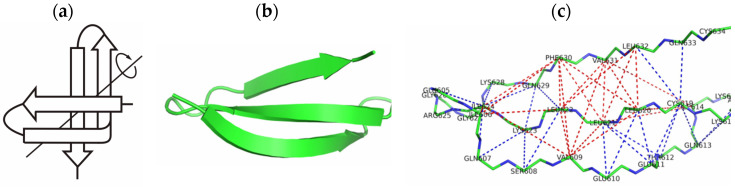
A schematic representation of the 3β-corner (**a**), ribbon and wireframe model of the 3β-corner, (**b**,**c**) beta-adrenergic receptor kinase 1 (bos taurus), PDB ID 1OMW region: A586-A625. Putative hydrophobic contacts are highlighted by dashed red lines, and hydrogen bonds by dashed blue lines.

The 3β-corner can be represented as a triple-stranded β-sheet folded on to itself so that two ββ-hairpins are packed roughly orthogonally in different layers and the central strand bends by ≈90° in right-handed direction when passing from one β-layer to the other [11] (Figure 1). All the 3β-corners observed in proteins can be considered as Z-like β-sheets when viewed from their concave surfaces.

The molecular dynamics experiment provides a range of tools for studying the properties of SSSs and their stability, and, consequently, it is crucial for the selection of candidate protein structures as folding nuclei [12].

## 2. Results

### 2.1. The 3β-Corner Motif as an Autonomous Structure

In the first stage, we examined the possibility of studying the SSS type 3β-corner as autonomously stable in an aqueous medium, that is, outside the protein globule. In this study, autonomous stability refers to the preservation of the structural topology of the 3β-corner in the MD experiment for the whole protein and separately from the protein environment in an aqueous medium. To do this, we removed parts of the structures corresponding to the 3β-corners from the standard files in the “.*pdb” format extracted from the PDB bank.

We carried out a comparative analysis of changes in the geometric characteristics of the 3β-corners in the composition of whole proteins and autonomously in an aqueous medium on the basis of the results of a 300 ns MD experiment. We performed this comparative analysis on two proteins, PDB ID 2E3H and PDB ID 2E3I.

Analyzing the results of the MD experiment revealed the strict preservation of the 3β-corner geometry in the protein and outside the protein (Table 1). The geometric characteristics were obtained by clustering the MD trajectory corresponding to the values for the conformer in the major cluster. By major cluster, we mean the conformer of the structure under study, which inhabits the trajectory for at least 80% of the total duration of the analysis. Table 1 presents the calculated values of the characteristics of the 3β-corner in the course of the MD experiments carried out with an individual SSS and the whole protein, where this 3β-corner is its constituent block. As an example, the table lists the characteristics of the two 3β-corners recognized in two proteins: 2E3H, chain A, region: 216–246 and PDB ID 2E3I, chain A, region: A62–A93 (Table S1).

The values of the solvent-accessible area, gyration radius, and number of hydrogen bonds of the 3β-corner structure were determined before the start of the experiment (experimental structure from the PDB bank), and their changes throughout the course of the experiment were analyzed. Table 1 illustrates the values of the studied parameters for the major cluster of the 3β-corner that participates in the experiment autonomously (outside the protein molecule) and as a part of the protein. The table lists the number of hydrogen bonds. For example, for 2E3I in the initial structure, 14 were found. Table 1 lists the RMSD values calculated for these structures during the MD experiment. For example, the following were observed for 2E3H:

- between the initial structure (PDB) and the structure outside the protein, it was equal to 2.8 Å;
- between the initial structure and the structure in the composition of the protein, it was equal to 5.6 Å.

These RMSD values were relatively small and favored the stability of the 3β-corner outside the protein molecule. The values of the studied characteristics 2E3I, chain A, region: A62–A93 also speak in favor of the stability of the 3β-corner outside the protein molecule.

**Figure 2:**
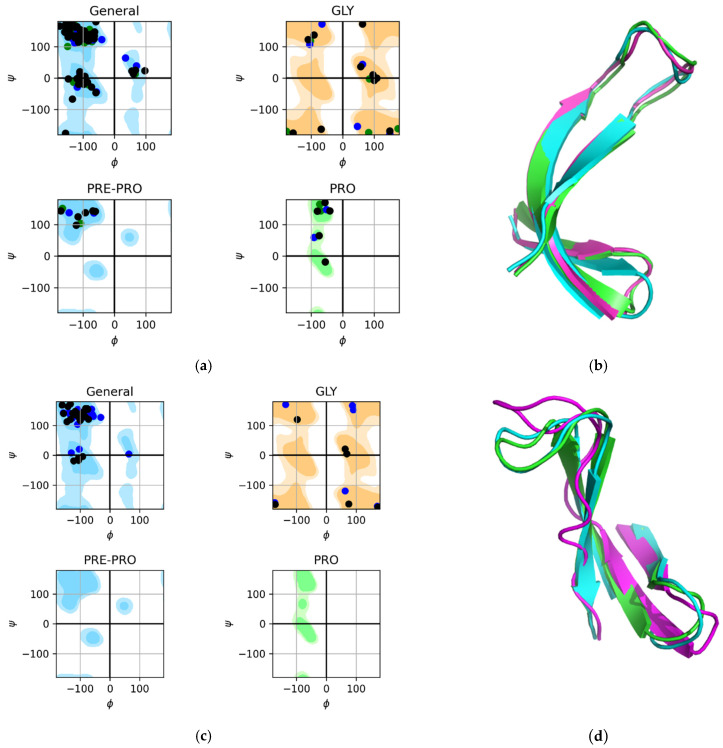
Distribution of angles φ and ψ on the Ramachandran map of the studied amino acid residues of the 3β-corners of 2E3H, chain A, region: 216–246 and 2E3I, chain A, region: A62–A93 during the MD experiment. The calculated angles are highlighted: for the initial structure, in yellow; the angles for the major cluster of the 3β-corner in the protein composition, in green; and the angles for the major cluster of the 3β-corner that participated autonomously in the experiment, in black. (**a**) Ramachandran map for all amino acid residues (except for amino acids highlighted on a separate map) and glycine GLY for 2E3H. (**b**) Schematic representation of the 2E3I of the original motif (green), the major cluster of the 3β-corner within the protein (red), and the major cluster of the 3β-corner that participated autonomously in the experiment (grey). (**c**) Ramachandran map for all amino acid residues. GLY, amino acid before proline; Pre-PRO, proline PRO for 2E3H. (**d**) Schematic representation of the 2E3I of the motif (green), the major cluster of the 3β-corner within the protein (red), and the major cluster of the 3β-corner that participated autonomously in the experiment (grey).

To check the stability of the studied 3β-corners in the course of a computational experiment, we recorded the values of the torsion angles of each amino acid residue of the structure to analyze the change in conformation. According to the distribution of the limiting values of the angles φ and ψ on the Ramachandran map for all possible regions, a conformational description of all the remains of the studied structures was performed. Figure 2 shows the distributions of the values of the angles φ and ψ on the Ramachandran map of the studied amino acids. 3β-corners 2E3H and 2E3I were used during the MD experiment. The drawing contains two cards: Ramachandran plot for all amino acid motives, and for glycine. Green dots are the calculated angles for the initial structure (PDB), yellow dots are the calculated angles for the major cluster of the 3β-corner in the protein, and black dots are the angles for the major cluster of the 3β-corner that participated in the autonomous experiment. Since the amino acid sequence of the motif under study does not contain proline, the analysis of the allowed proline regions was excluded. From the figure, we see that the investigated amino acid residues in the 3β-corner did not leave the allowed areas. The angles of the studied amino acids for the original structure, the major cluster of the 3β-corner in the protein composition, and the major cluster of the 3β-corner that participated in the experiment autonomously were always located close to each other. All these peaks (as well as other parameters analyzed in the MD experiment) also favored the stability of this structure.

Figure 2b,d illustrates the overlay of images of the original motif, major cluster of the 3β-corner within the protein, and major cluster of the 3β-corner that participated in the experiment outside the protein (autonomously). We could clearly see the complete coincidence of the elements of the secondary structure, β-strands. Minimal deviation of irregular areas (constrictions) was observed between the three analyzed images of the studied 3β-corner. However, the magnitude of the deviation, that is, the distance between the irregular sections of the structures, was insignificant. This fact, established during the experiment, proved that this super-secondary structure is a stable autonomous structure in aquatic environments. Ramachandran maps showed the distribution of the values of the angles φ and ψ for the studied amino acid residues of two types of the 3β-angle during the MD experiment. We can state that most of the studied amino acids for both studied structures, for major clusters of 3β-corners in the composition of the protein and for major clusters of autonomous 3β-corners during the experiment, had angles φ and ψ close in value. This behavior of the 3β-corner structures is typical and not limited to the cases described.

In the second stage, we examined the possibility of reducing the duration of the MD experiment for autonomous 3β-corners in an aqueous medium. A comparative analysis was conducted on the behavior of the structures of the 3β-corners in a “long” trajectory (300 ns) and a “short” trajectory (20 ns). This methodology was guided by several arguments to reduce the duration of MD. Throughout most of our experiments, we observed that clustering the MD trajectory allowed us to clearly identify the conformer that inhabits the trajectory at least 80% of the time. In the MD experiment, we did not observe any significant changes in the values of the geometric characteristics for the 3β-angles between the “long” and “short” trajectories. A justified reduction in MD time makes it possible to significantly improve calculation results.

**Figure 3:**
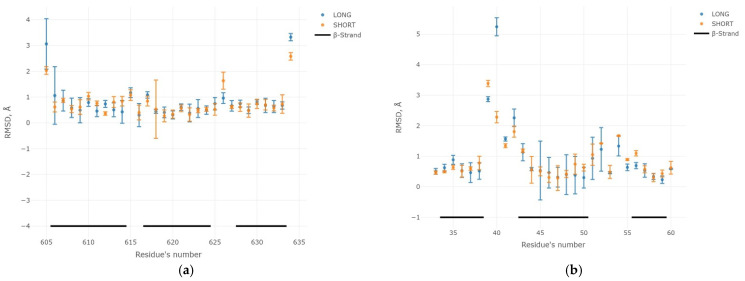

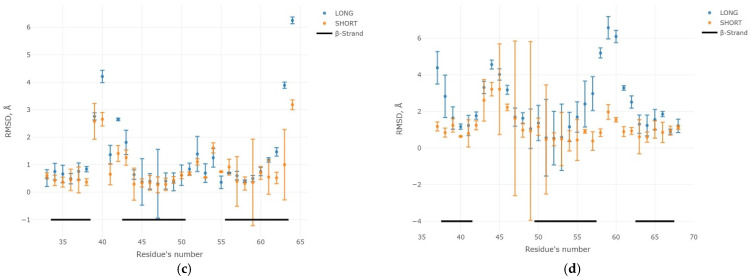
Comparison of changes in the values of the RMSD along the “long” (red color) and “short” (blue color) MDs. Unstructured sections of polypeptide chain are marked with omissions, whereas β-strands correspond to black lines at the bottom of each panel. Averages value of RMSD and associated standard deviations. Typical cases are presented for structures of the 3β-corner type: 1OMV: region A605–A634 (**a**), 2CX8: region A33–A60 (**b**), 1V6Z: region A33–A64 (**c**), and 2BJQ region: A32–A62 (**d**). All changes were made in three technical repetitions.

Results of “long” and “short” MD for some instances of 3β-corner structures excised from 1OMV (PDB ID), 2CX8 (PDB ID), 1V6Z (PDB ID), and 2BJQ (PDB ID) proteins (Figure 3) showed that the lower values of RMSD in both “long” and “short” trajectory dynamics corresponds to sections of the amino acid sequences identified as β-strand (in Figure 3, black lines at the bottom). On the contrary, unstructured areas (Figure 3, blank region on the bottom) were subject to greater variability in MD experiments, which was the most prominent for 2BJQ region, A32-A62 (Figure 3d). Results with similar values of RMSD were observed in the “long” and “short” MD experiments (Table S2).

On the basis of the obtained results of the “long” and “short” MD, we assert that the 3β-corners in the composition of the studied set retained their stability in the aqueous environment. The optimal time for the MD experiments was 20 ns. Further reduction in the duration of MD was deemed inappropriate. The next section describes the MD results for the entire dataset.

### 2.2. Control Experiments

**Figure 4:**
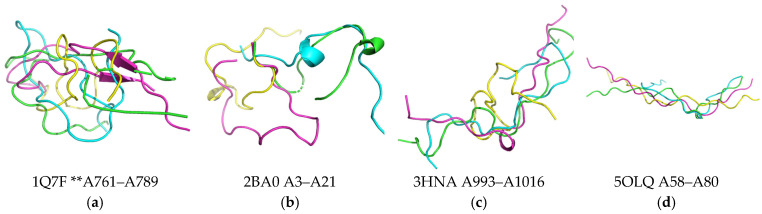
A set of overlapped protein structures used as a negative control. (**a**) Brain tumor protein (drosophila melanogaster) PDB ID 1Q7F region: A761–A789; (**b**) exosome complex component Rrp4 (archaeoglobus fulgidus) PDB ID 2BA0 region: A3–A21; (**c**) histone-lysine N-methyltransferase EHMT1 (homo sapiens) PDB ID 3HNA region: A993–A1016; (**d**) putative pectate lyase L (bacteroides thetaiotaomicron) PDB ID 5OLQ region: A58–A80. Color-scale legend: green color indicates structure in the protein composition (PDB data); blue, pink, and yellow colors define technical repetitions; **—chain A, region (PDB).

Control experiments were performed with the involvement of several types of protein structures close to 3β-corner structures in the length of amino acid sequence but were different in terms of spatial arrangement (Figure 4). It was demonstrated (Figure 4) that negative controls embodied structures that did not contain β-strands, as well as α-helix. The selected testing structures were characterized by radius of gyration and solvent-accessible areas comparable to the study set of 3β-corner structures (Table S3).

The MD experiments displayed changes in the geometry of negative control structures and structures in the 3β-corner dataset. The results of MD experiments in three technical repetitions (20 ns) revealed high variability of RMSD values and a low number of hydrogen bonds among the selected negative control structures, which defines them as unstructured tangles (Figure 4). Nevertheless, visualization before and after the MD experiment showed that the structures 1Q7F and 2BA0 arranged a small β-hairpin and one turn of the α-helix, respectively (according to STRIDE), only in one of the technical replications. Meanwhile, the gyration radius and solvent-accessible area were significantly smaller compared to those for the 3β-corner set (Table S3).

### 2.3. Results of the MD Experiment for 3β-Corners

**Figure 5:**
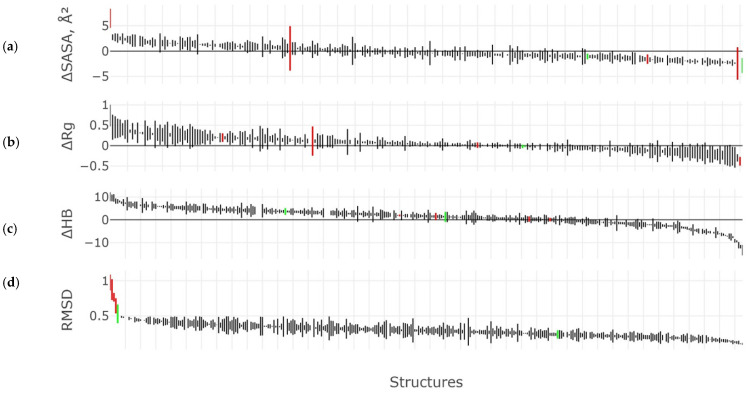
Comparative analysis of MD geometric characteristics for 330 structures of 3β-corners of experimental structures (exp) versus structures after MD (data are shown in three technical replicates). Distribution of (**a**) solvent-accessible surface area (SASA, Å^2^); (**b**) gyration radius (Rg); (**c**) the number of hydrogen bonds (HB); (**d**) RMSD (nm). All the structures involved in the experiment were deposited along the OH axis. The order of structures along the OH axis was generated separately for each characteristic and arranged in incremental order (from maximum to minimum). Red lines indicate measurements for the structures of the control set (negative control); green lines indicate measurements of selected motifs within a whole protein molecule; black lines designate measurements of characteristics for 3β-corners of the target dataset.

Comparative analysis was managed for 330 experimental PDB structures under the study, and following the MD operation, the number of hydrogen bonds, alterations of permitted and prohibited areas of the Ramachandran map (Figure 5), and solvent-accessible areas (the number of water molecules in the contact or residual water in Å^2^) were calculated.

Results of MD experiments for all structures examined in three technical repetitions are summarized in Figure 5. Variation of solvent-accessible area (Figure 5a) of the experimental 3β-corner structures during molecular dynamics was estimated as

ΔSASA = SASA (EXP) − SASA (MD)(1)

Whether or not the value was equal to zero along the OY axis (marked with a black line) indicated the unaltered solvent-accessible area of the structure during the MD experiment; thus, the indicator remained equal to that before the MD experiment (Equation (1). We noticed that most of the structures were localized close to a y~0 value, meaning a slight change of the measured parameter during MD modeling. However, even if the measured value was not equal to zero, most of structures lay along the selected axis (y = 0). The SASA values were calculated according to three technical specifications and represented by value spreads (Figure 5a, red lines), the largest of which were found among structures of the control set (negative control) suggesting instability of structures. At the same time, we can note that SASA had a minimal variation in the three techniques for the selected motifs that participated in the MD experiment as part of a protein molecule, which implied the stability of structures. Values of ∆SASA were characterized by a small spread for three technical repetitions among 3β-corner structures of the target dataset.

Similarly, most of the tested structures demonstrated a slight change of the radius of gyration (Equation (2)) through three replications of the MD experiment (Figure 5b).

∆Rg = Rg(EXP) − Rg(MD)(2)

The ∆Rg values of the control set structures were also characterized by a small spread, and the behavior of these structures for this indicator fell within the general scenario.

The number of hydrogen bonds (Equation (3)) is yet another important characteristic of the studied structures. The number of hydrogen bonds and their quantitative change were determined for each structure through the MD experiment (Figure 5c).

∆H_B = H_B (EXP) − H_B (MD)(3)

The zero value on the OY axis (y = 0) means the lack of quantitative changes of hydrogen bonds during the experiment. Most of the structures lost the minimum number of hydrogen bonds during the MD experiment, suggesting the stability of structures. Moreover, there was minimal spread of ∆HB through over technical replicates of MD in the majority of tested structures assuming high stability of the 3β-corners (Figure 5c). The amount of hydrogen bonds broken among the studied structures reached 10 and ranged from 0% to 50% of the total amount of bonds established before the experiment. The ∆HB spread was insignificant in the negative control set, probably because structures of the control set did not have a large number of hydrogen bonds initially; thence, such protein blocks did not have a hydrophobic core stabilizing the structure.

The mean RMSD value for three replicates of MD experiments varied roughly less than 5 Å among the structures of the target dataset in each single case. This confirmed the hypothesis that the 3β-corner is a stable structure. On the contrary, structures of the negative control set showed wider spread of RMSD, significantly exceeding out of 5 Å and reaching up to 10 Å, suggesting low stability of the control set structures.

More detailed information regarding results of the MD testing under the studied structures can be approached in Tables S2 and S4. Specifically, the radius of gyration, solvent-accessible area, the number of hydrogen bonds, and retention of amino acid residue position at a certain zone of the Ramachandran map for each of the examined 330 structural motifs were calculated and collected (Table S4). These data were associated with coordinates of localization of motifs (3β-corners) being found in tested proteins and with an average value of RMSD for such motifs. It was found that only 18 3β-corner structures out of 330 bore the calculated average RMSD values for three MD techniques more than 5 Å, whereas 50 structures were characterized by RMSD values less than 2 Å, and the rest of the structures encompassed a range between 2 and 5 Å (Table S4). The narrow range of calculated values evidenced the stability of 3ß-corner turning a fresh look at this structure and to consider it as an independent object of research in the field of structural biology. On the contrary, structures of the control set were featured by greater variability of calculated parameters.

**Figure 6:**
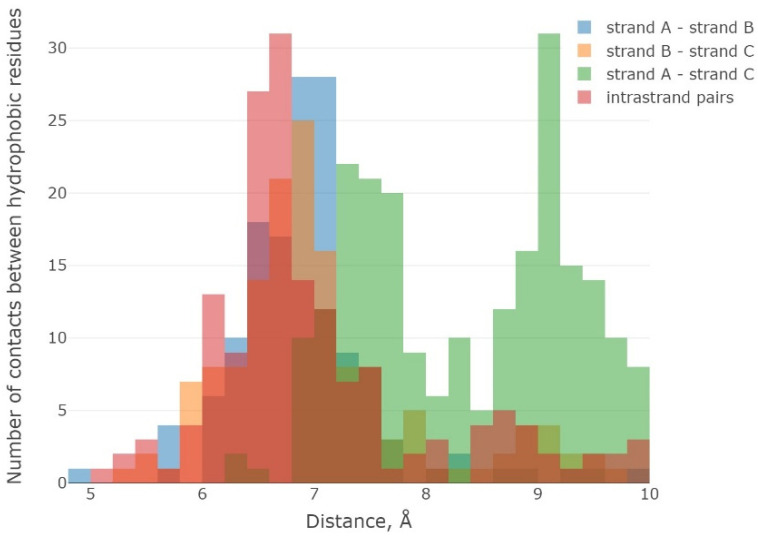
Distribution of distances between interacting hydrophobic amino acid residues in 3ß-corners: within one β-strand (“inside-strand”) and between different β-strands (“cross-strand”): red color indicates contacts within one strand, blue color indicates contacts of hydrophobic amino acid residues between β-strands A and B, yellow color designates contacts between β-strands B and C, and green color specifies contacts between β-strands A and C.

The beguiling result was achieved next to the analysis of probable contacts between amino acid residues involved in the organization and stabilization of the hydrophobic core of 3β-corners. Indeed, we observed that hydrophobic amino acid residues hold about 40% (Table S5) of the total amount of residues in the selected set of structures. Analysis of probable contacts between amino acid residues revealed a bimodal distribution of distances between interacting hydrophobic amino acids (Figure 6).

The number of contacts arranged within one β-strand (inside-strand) was only a part of the likely contacts (Figure 6, red color), while most of the contacts were attributed to interactions of amino acid residues localized in different β-strands (“cross-strand”).

There were two upper limits in the distribution of distances between interacting hydrophobic amino acid residues in the 3β-corners that can be determined: (1) the “inside-strand” type interactions and (2) contacts between adjacent β-strands in the amino acid sequence (A → B, B → C, and A → C). The maximum distribution of distances between contacting amino acids was at 6,8–7 Å and the second maximum was at 9.4–9.6 Å. It should be noticed that the distance 9.4–9.6 Å was characteristic of contacts between the first and the third β-strands (A → C) of 3β-corners since these elements of SSSs (by definition) were localized on different orthogonal planes, so the distances between them were greater. Thus, the 3β-corner structures were distinguished by a high proportion of hydrophobic amino acid residues and “cross-strand”-type contacts, unique compact stacking, and autonomous stability in a molecular dynamic experiment.

## 3. Discussion

Research on SSSs is important due to unique and compact spatial packing of the polypeptide chain, which makes it possible to consider SSSs as possible nuclei for protein folding. Studies on the modeling of protein structures and stability of the obtained proteins have been conducted using molecular dynamics simulation experiments [13,14]. The present study analyzed the autonomous stability of the 3β-corner-type structural motif in an aqueous medium outside the protein environment. The MD study of the structural motif, which is small (in terms of the number of atoms) compared to the entire protein molecule, is characterized by high performance, relatively low computational requirements, and low experimental costs.

This study aimed to substantiate the possibility of studying the structure of the 3β-corner type outside the protein globule. To do so, we selected a set of 3β-corners of 330 structures extracted from the PDB bank. The generated structure database facilitated a comprehensive study of the characteristics of the motif when examining a larger set.

We conducted molecular dynamics experiments to establish the autonomous stability of a super-secondary structure of the 3β-corner type outside the protein environment. The stability of conformational templates was analyzed on the basis of the distribution of the angles φ and ψ on the Ramachandran map during the experiment. A conformational description of the amino acid residues of the studied structures was performed according to the distribution of the limiting values of the angles φ and ψ on the Ramachandran map for all possible regions. The change in the conformation of the structure of the motif under the study was analyzed in the molecular dynamics experiment by recording the frequency of hitting the torsion angles of amino acid residues in certain areas.

In the study, we determined that the structures of 3β-corners behave in the same way in the MD experiment. Thus, analysis of the changes in geometric characteristics did not reveal significant differences between the experimental structures extracted from the PDB bank and the autonomous structures of the 3β-corners after MD operation. On the basis of the obtained results, we recommend an MD duration of 20 ns.

## 4. Materials and Methods

### 4.1. Dataset of 3β-Corner Structures

In this study, we focused on a simple type of SSS—3β-corner. The aim of this study was to analyze the stability of 3β-corner as an autonomous unit or outside the protein globule in an aqueous environment. A set of 330 structures of the 3β-corner type was selected from the Protein Data Bank (PDB) (https://www.rcsb.org/, accessed on 5 August 2022) to perform MD experiments with the following analysis of standard deviation, change in gyration radius, solvent-accessible area, lifetime of the major conformer and torsion angles, and the number of hydrogen bonds reported to characterize the stability of proteins. Super-secondary 3β-corner structures are widely distributed, and there are a few small proteins consisting of only the 3β-corner and short irregular regions in nature [15,16].

**Figure 7:**
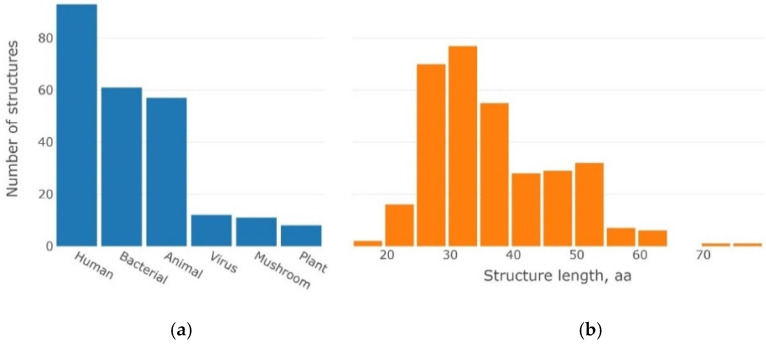
Origin of proteins in which 3β-corners were identified (**a**); length distribution of 3β-corners in the examined dataset (**b**).

The collected dataset was organized from β-proteins, most of which contained small β-barrels according to the SCOP [17] classification (https://scop.berkeley.edu/, accessed on 5 August 2022) folds b.34 (SH3-like; 21 superfamilies), b.43 (common domain of reductase/isomerase/elongation factor; three superfamilies), b.47 (trypsin-like serine proteases; one superfamily), b.55 (domain-like PH; one superfamily), etc. (see Table S6) [17,18,19]. Small stacks of β-barrel represent a closed structure, wherein the first and last β-weights were stabilized by hydrogen bonds [20]. The β-barrel structures can also be represented as two orthogonally arranged β-sheets [21]. Such β-barrel structures are characterized by a few β-strands (n = 3–5) and an elliptical type of cross-section with high shear values (S~6–10), which provide a tight fit—a “flattened” ellipse. The 3β-corner structural motifs ordered as “β-Strand → Coil → β-Strand → Coil → β-Strand” were extracted from the β-barrel folds. The correlation of amino acid residues within the selected structures to elements of the secondary structure was elaborated by using three STRIDE algorithms [22], DSSP [23], and iCn3D [24]. The results of convergence between three algorithms utilized for the identification of elements of the secondary structure are presented in Table S7. The match rate between STRIDE/DSSP, Stride/iCn3D, and DSSP/iCn3D was approximately 50% (Table S7). β-Strands in the 3β-corner structure are usually short and consist of 4–6 to 10 amino acid residues, connected by loops. Short loops (from three to seven amino acid residues) shape a turn, while long loops (8–12 amino acid residues) are characterized by an unstructured shape. The studied dataset was represented by motifs extracted from proteins of various origins and containing different lengths of its constituent elements (Figure 7 and Table S4).

SSSs were selected for homologous and non-homologous proteins of various origins (Figure 7a). Most of these proteins belong to humans, animals, and bacteria. There are also small groups of plant and viral proteins (Figure 7a). This observation is consistent with the variety of annotated protein structures in the PDB for different entities.

The analysis of lengths amongst selected 3β-corners showed that the majority of studied structures were 25–40 amino acids in length. Moreover, depending on the length, the maximum distribution of 3β-corners fell on 30–35 amino acid residues (Figure 7b), and only a few of the 3β-corners consisted of 45–55 amino acid residues.

The gathered 3β-corners were extracted from the PDB and SCOP and are presented in Table S6.

### 4.2. Molecular Dynamics Simulation

Simulations were performed using GROMACS software package (version 2021.4). The simulation process was identical for every simulation except simulation time (20 or 300 ns) and some parameters, depending on size and charge of every specific molecule, and was automatically calculated. Configuration and procedure already were used in our previous studies [25,26,27] but were modified for current research. Molecules were prepared with pdb2gmx tool (GROMACS, version 2021.4), which adds missing hydrogen atoms and forms the correct topology system using CHARMM36 force field converted for GROMACS. Molecules were placed into rectangular boxes for simulation. Box size was automatically configured (with gmx editconf tool) for each simulation depending on protein size, leaving at least 1.0 nm between molecule and box borders.

Boxes were also filled with water (gmx solvate tool, GROMACS, version 2021.4). Water was represented with SPC model selected as a compromise between simulation performance and realism of behavior in normal conditions (see [28] for details about water model comparison). The simulation system also was neutralized with Na+ or Cl^−^ ions. GROMACS automatically calculated the charge of the system and replaced some amount of water molecules in the box with ions to make the system neutral (using gmx genion tool). If the charge of the system was negative, it added positive ions (sodium), and if the system’s charge was positive, it added negative (chlorine) ions.

The next preparation step was energy minimization (EM). EM is short specific simulation performed using the steepest decent algorithm. The simulation was performed until energy became lower than 1000.0 kJ/mol/nm or until 1000 simulation steps (0.002 ps per step [29]).

The final preparation was heating pre-run: the simulation system was heated from 5 to 311 K for 200 ps with 0.001 step using a v-rescale temperature coupling algorithm. The molecular dynamics simulation itself ran for 20 or 300 ns (different runs had different simulation times, which is mentioned in the corresponding sections of the study). Temperature for simulation was kept at 311 K. The simulation was performed by GROMACS default leap-frog algorithm for integrating Newton’s equations of motion. Simulation step was 0.002 ps.

Resulting trajectories were processed using GROMACS analysis utilities such as gmx rms, gmx gyrate, and gmx sasa. Clusterization was performed using the gmx clusters utilized. Cut-off value for clusterization was a varying value depending on the first clusterisation result with a cut-off = 0.3 nm: if the result contained > 12 clusters, clusterization was redone with a larger cut-off value until the cluster amount reached 20. Moreover, if there were < 8 clusters, clusterization was redone with a larger cutoff. The step for raising/lowering cutoff was set to 0.01 nm, and the limit for the clusterization attempt was set to 30.

RMSD changes were calculated for the backbone for all the trajectories after the heating step. These data in XG format were aggregated with our own specially written scripts. Intact values were taken from an initial PDB file or from the first step of the trajectory. MD resulting values were the average of values for trajectory parts where structure conformation matched the major cluster in the final clusterization result.

All the GROMACS configuration files are available on the following link https://github.com/protdb/beta-corner-stability/tree/master/GROMACS%20configuration (accessed on 5 August 2022).

A total of 330 structures were used in the MD experiments. In this study, the following MD experiments were carried out for SSS of the 3β-corner type:

- “long” dynamics, simulation duration 300 ns;
- “short” dynamics, simulation duration 20 ns;
- part of the whole protein, simulation duration of 300 ns.

According to the MD results, the changes in the following geometric characteristics of the SSS were analyzed:

- root-mean-square deviation (RMSD) and gyration radius change (Rg);
- solvent accessible surface area (SASA);
- lifetime of major conformer;
- change in torsion angles;
- change in the number of hydrogen bonds.

Data on the conditions of MD experiments are available on the following link https://github.com/protdb/beta-corner-stability (accessed on 5 August 2022).

## 5. Conclusions

It can be concluded that in the water environment, the 3β-corner is rather stable per se, i.e., without the rest part of a protein molecule, and it can act as the nucleus or “ready-made” building block in protein folding. The 3β-corner can also be considered as an independent object for study in field of structural biology.

Understanding the protein architecture and recognizing and studying their individual structural blocks with unique and compact polypeptide chain folds provides an opportunity to gain insight regarding the structure, geometry, internal contacts, and patterns of organization of structural motifs of protein molecules. This knowledge provides a strong basis for addressing fundamental studies, such as prediction and modeling of three-dimensional protein structures, protein folding, and structural classification of proteins, as well as applied problems such as developing novel approaches for disease diagnosis and improving our understanding of pathogenesis, identifying drug targets, and designing proteins with desired properties (mimetics).

We annotated a set of 3β-corner SSSs in 330 structures. As a result of the MD experiments, we showed the autonomous stability of this type of super-secondary structure and demonstrated the possibility of analyzing them as independent objects. We observed the preservation of key geometric characteristics because of the MD of autonomous structures of 3β-corners in comparison with experimental structures extracted from the PDB. We present an annotated set of 3β-corners of the research in the fields of structural biology and biomedicine.

## Figures and Tables

**Table 1:** Comparative analysis of the geometric characteristics of the 3β-corner in a protein environment and autonomously from protein in an aqueous medium (three technical repetitions).

Structure	SASA ± SD (Å^2^)	R_g_ ± SD	B_H_ ± SD	RMSD ± SD (Å)
PDB ID 2E3H, chain A, region: 216–246				
PDB structure *	3202.64	1.3	18	–
MD: 3β-corner **	3061.3 ± 150.4	1.23 ± 0.3	14 ± 1	2.77 ± 0.5
MD: whole protein ***	3606.075 ± 13.499	1.3 ± 0.04	16 ± 0	6.67 ± 0.72
PDB ID 2E3I, chain A, region: A62–A93.				
PDB structure	3335.75	1.2	22	–
MD: 3β-corner	3085.9 ± 130.3	1.19 ± 0.1	14 ± 1	2.1 ± 0.9
MD: whole protein	3846.33 ± 4.59	1.23 ± 0.01	19 ± 2	6.86 ± 0.37

Designations: SASA is the area available for the solvent, Å^2^; Rg is the gyration radius; B_H_ is the hydrogen bond; and PDB structure * is the experimental structure extracted from the PDB bank. Values are given for the conformer in the major cluster of the MD trajectory. Values are given for the structure of the 3β-corner in the protein; MD: 3β-corner **—results of MD experiment for autonomous 3β-corner outside the protein globule; MD: whole protein ***—MD experiment of a whole protein containing a 3β-corner; the results are shown for the whole protein (three technical repetitions).

## Data Availability

Not applicable.

## Supplementary Materials

The following supporting information can be downloaded at https://www.mdpi.com/article/10.3390/ijms231911674/s1.
